# Prosocial Behavior in Adolescence: Gender Differences in Development and Links with Empathy

**DOI:** 10.1007/s10964-017-0786-1

**Published:** 2017-11-28

**Authors:** Jolien Van der Graaff, Gustavo Carlo, Elisabetta Crocetti, Hans M. Koot, Susan Branje

**Affiliations:** 1 Research Centre Adolescent Development, Utrecht University, Utrecht, The Netherlands; 2 Department of Human Development and Family Science, University of Missouri, Missouri, USA; 3 Department of Psychology, Alma Mater Studiorum Università di Bologna, Bologna, Italy; 4 Department of Clinical Neuro and Developmental Psychology, Vrije Universiteit Amsterdam, Amsterdam, The Netherlands

**Keywords:** Prosocial behavior, Perspective taking, Empathic concern, Adolescence, Development

## Abstract

Although adolescents’ prosocial behavior is related to various positive outcomes, longitudinal research on its development and predictors is still sparse. This 6-wave longitudinal study investigated the development of prosocial behavior across adolescence, and examined longitudinal associations with perspective taking and empathic concern. Participants were 497 adolescents (*M*_age t1 = _13.03 years, 43% girls) who reported on their prosocial behaviors, empathic concern, and perspective taking. The results revealed marked gender differences in the development of prosocial behavior. For boys, levels of prosocial behavior were stable until age 14, followed by an increase until age 17, and a slight decrease thereafter. For girls, prosocial behavior increased until age 16 years and then slightly decreased. Regarding longitudinal associations, empathic concern was consistently related to subsequent prosocial behavior. However, perspective taking was only indirectly related to prosocial behavior, via its effect on empathic concern. Tests of the direction of effects showed support for the notion that earlier prosocial behavior predicts subsequent empathy-related traits, but only for girls. The findings support cognitive-developmental and moral socialization theories of prosocial development and the primary role of moral emotions in predicting prosocial behaviors. Our findings inform strategies to foster prosocial behaviors by emphasizing moral *emotions* rather than moral *cognitions* during adolescence.

## Introduction

Adolescents’ prosocial behavior, or voluntary behavior intended to benefit others (Eisenberg, Fabes, & Spinrad, 2006), has been linked with several positive outcomes, including high self-esteem, academic success, and high quality relationships (Laible et al. 2004; Padilla-Walker and Carlo 2014; Wentzel 1993). Although previous studies have consistently shown prosocial behavior to increase during early childhood (see Eisenberg et al. 1998), research on the development of prosocial behavior during adolescence has revealed conflicting results (Carlo et al. 2007, 2015; Eisenberg et al. 2005; Luengo Kanacri et al. 2013). Regarding potential predictors of prosocial behavior, empathy is thought to provide the motivation to express helping behavior. Both the understanding of others’ inner states (i.e., perspective taking) and the experience of feelings of concern for others (i.e., empathic concern) are believed to facilitate prosocial behavior (Batson 1991; Hoffman 2000). Conversely, engaging in prosocial behavior may also foster adolescents’ tendency to exhibit perspective taking and empathic concern (Carlo et al. 2015). However, no previous studies have investigated the longitudinal links of both perspective taking and empathic concern with prosocial behavior (and vice versa) across adolescence. Therefore, this 6-wave study, first, investigated the development of prosocial behavior from age 13 to 18 years and, second, examined the longitudinal links between perspective taking, empathic concern and prosocial behavior. In addition, since prior research suggests that gender differences may exist both in the development (e.g., Carlo et al. 2007; Van der Graaff et al. 2014) and the prediction of prosocial tendencies (e.g., Caravita et al. 2009), we tested for gender differences in all analyses.

### Development of Prosocial Behavior

Although prosocial development has long been studied, and general age-related increases have been reported from infancy through early adulthood (see Eisenberg et al. 1998; Crocetti et al. 2016), only a few longitudinal studies have examined changes in prosocial behavior across a broad age range in adolescence (i.e., Carlo et al. 2015; Luengo Kanacri et al. 2013). Yet, there is considerable evidence that several physical, cognitive, and relational changes occur during adolescence that impact social functioning. First, adolescents’ physical maturity and increasing autonomy may allow them to engage in a wider variety of prosocial actions (Carlo et al. 2012; Fabes et al. 1999). Second, advances in perspective taking (e.g., Van der Graaff et al. 2014) may facilitate higher-stage moral reasoning, which in turn should promote prosocial behavior (Blasi 1980; Eisenberg and Spinrad 2014; Kohlberg 1969). Third, increased frequency of peer interactions and interest in intimate and romantic relationships develop alongside an increase in social competence (Steinberg and Morris 2001) and may also foster adolescents’ other-oriented behavior (Fabes et al. 1999; Wentzel 2014). However, other changes during adolescence may negatively impact the development of adolescents’ prosocial tendencies. For instance, changes in affective processing and brain maturation might challenge emotion regulation in mid-adolescence (see Crone and Dahl 2012), which may temporarily diminish adolescents’ ability to direct their attention to others’ emotional needs and therefore decrease prosocial tendencies (e.g., Eisenberg et al. 1996, 2000; Padilla‐Walker and Christensen 2011). Thus, conceptually, mean levels of prosocial behavior can be expected either to increase during adolescence or to show a temporary decrease.

Moreover, the development of prosocial behavior may be different for boys and girls. According to gender socialization theorists, girls are socialized to show nurturance and caring, whereas boys are socialized to inhibit these kinds of prosocial behavior (Brody 1999). During adolescence, gender-specific socialization pressures are thought to strengthen and boys and girls may increasingly adhere to gender stereotypes (Alfieri et al. 1996; Hill and Lynch 1983), which may result in gender-specific developmental trends in prosocial behavior. Moreover, previous research revealed gender specific developmental trends in moral reasoning (Eisenberg et al 1991), empathic concern and perspective taking (e.g., Carlo et al. 2015; Van der Graaff et al. 2014). Given the conceptual connection between these constructs and prosocial behavior (e.g., Hoffman 2000; Staub 1978), it is important to investigate gender differences in the development of prosocial behavior as well.

Results from the few previous longitudinal studies on prosocial development in adolescence are inconclusive. Whereas increases were found in prosocial behavior towards strangers between age 13 and 16 (Carlo et al. 2015), and in helping behavior between age 15 and 18 (Eisenberg et al. 2005), other studies found non-linear growth between age 12 and 14 (Caprara et al. 2015), stable levels in self-reported prosocial behavior between age 10 and 14 (Nantel‐Vivier et al. 2009), and even decreases between age 13 and 18 (Carlo et al. 2007; Luengo Kanacri et al. 2013). Regarding gender differences, all of these studies revealed boys to report lower levels of prosocial behavior than girls, but the issue of potential gender differences in developmental patterns has received surprisingly little attention. Only two of the studies investigated gender moderation, of which one revealed no significant gender moderation (Carlo et al. 2015) but the other found a decrease in prosocial behavior that was stronger for boys than for girls (Carlo et al. 2007).

Given the inconsistencies in the literature, and the relative dearth of comprehensive studies on this topic, the aim of the current study is to expand our understanding of prosocial development in adolescence. To our knowledge, this six-wave longitudinal study is the first to investigate age trends and gender differences from early to late adolescence (i.e., between ages 13–18 years). The comprehensive design of the current study allows for a thorough investigation of potentially complex and gender-specific growth patterns, which may help explain inconsistencies between previous studies.

### Longitudinal Links between Empathic Concern, Perspective Taking, and Prosocial Behavior

Empathy is generally deemed a multidimensional construct, involving affective as well as cognitive processes (see Davis 1996; Decety and Jackson 2004). Affective empathy refers to the vicarious experience of emotions consistent with those of the observed person and often results in empathic concern, which involves feelings of sorrow or concern for another. Cognitive empathy, or perspective taking, can be defined as the awareness and understanding of another’s emotion (Davis 1983). A previous study on the mean-level development of empathic concern and perspective taking showed that both traits are still subject to change during adolescence (Van der Graaff et al. 2014). Empathic concern and perspective taking may both facilitate prosocial behavior (Batson 1991; Hoffman 2000), although there is some debate about the relative importance of “feeling” vs. “understanding” in predicting such actions, and longitudinal studies looking at the role of both empathic concern and perspective taking in prosocial behavior are scarce.

Regarding empathic concern, feelings of sorrow for someone else are thought to be an important motivation to alleviate others’ distress, and thus, to show helping or caring behavior towards others (Batson 1991; Batson et al. 1989; Eisenberg and Miller 1987). Previous research provides empirical support for a positive association between adolescents’ empathic concern and prosocial behavior, although the evidence mainly comes from cross-sectional studies (e.g., Berger et al. 2015; Caravita et al. 2009; Eisenberg and Miller 1987; Eisenberg et al. 2001). However, a recent study showed empathic concern also to predict prosocial behavior 1 year later during early to middle adolescence (Carlo et al. 2015).

Regarding perspective taking, individuals who have a high tendency to imagine the other’s psychological point of view are likely to be other-oriented and to be aware of others’ needs. Therefore, they can be expected to be better at finding ways to help others than are individuals low in perspective taking (Eisenberg et al. 2015). However, it has been suggested that although perspective taking may facilitate positive behavior, it can also be used to manipulate or take advantage of others (Hawley 2003; Sutton et al. 1999). Thus, perspective taking, in and of itself, may not directly predict prosocial behavior. However, instead perspective taking may affect prosocial behavior indirectly through empathic concern. That is, individuals who tend to take others’ perspectives become more likely to experience feelings of concern for those others and may subsequently show prosocial behavior (Batson et al. 1989; Eisenberg et al. 2001), although a previous study (using latent variables) showed that perspective taking did not predict empathic concern between ages 14 and 17 years (Van Lissa et al. 2014). Results of previous empirical studies on the link between perspective taking and prosocial behavior are indeed mixed (see Carlo et al. 2010a, for a meta-analytic review). For instance, whereas a cross-sectional study revealed no significant association between perspective taking and defending bully victims (Caravita et al. 2009), a longitudinal study revealed that higher levels of perspective taking did predict a higher willingness to intervene in bullying (Espelage et al. 2012). Further, higher perspective taking was directly related to higher prosocial behavior (Carlo et al. 2010b), and adolescents high on prosocial behavior were found to score high on both perspective taking and empathic concern (Berger et al. 2015). However, another cross-sectional study showed the association between perspective taking and prosocial behavior to be indirect through empathic concern rather than direct (Eisenberg et al. 2001).

Taken together, there is consistent support for empathic concern as a predictor of prosocial behavior, although evidence mainly comes from cross-sectional research. However, regarding the role of perspective taking in adolescents’ prosocial behavior both the theoretical and empirical literature is mixed. Therefore, this longitudinal study aims to clarify how empathic concern and perspective taking are related to prosocial behavior throughout adolescence.

### Prosocial Behavior Predicting Empathic Concern and Perspective Taking

Although previous studies have mainly focused on empathic concern and perspective taking as predictors of prosocial behavior, it is likely that the associations are bidirectional. First, engaging in prosocial behaviors provides adolescents with opportunities to show concern for others and to take others’ perspectives (Malti et al. 2009). Second, prosocial actions often evoke positive feedback from adults and peers, which may strengthen adolescents’ image of themselves as a caring and understanding person, and may reinforce them to behave accordingly (Carlo and Randall 2001; Crocetti et al. 2016). Indeed, the possible reciprocal relations between prosocial behavior, emotions, and cognitions likely result in a more integrated sense of moral self, which may account for strong moral identity (Carlo et al. 2015; Hardy and Carlo 2005). Despite these conceptual foundations, the few previous studies that examine reciprocal effects of prosocial behavior on empathy have not included perspective taking, though they do provide initial support for reciprocal relations between prosocial behavior and empathic concern (Carlo et al. 2015; Eisenberg et al. 1999). Thus, the current study is the first to investigate bidirectional relations across adolescence in the links among prosocial behavior and both empathic concern and perspective taking.

### Gender Differences in Longitudinal Links

As noted previously, gender and moral socialization theorists posit gender specific socialization experiences that orient girls towards nurturing, expressive, and caring behaviors. In contrast, boys are typically socialized towards masculine-typed behaviors that include instrumentality, assertion, and competitiveness (Eagly and Crowley 1986; Leaper 2015). Gender stereotypes and gender-specific socialization practices may not only result in differences in mean levels of prosocial behavior, but may also affect its links with empathic concern and perspective taking. For instance, previous research suggests that the cognitive process of perspective taking is a stronger motivator to show prosocial behavior for boys, whereas empathic concern may play a more important role in girls’ prosocial behavior (Eisenberg et al. 2001). Moreover, girls may receive more positive feedback when engaging in prosocial behavior than boys (Brody 1999; Eisenberg et al. 2006), which may result in stronger predictive effects of prosocial behavior on perspective taking and empathic concern for girls. Although previous studies provide some support for gender differences in the associations between perspective taking, empathic concern and prosocial behavior (Caravita et al. 2009; Eisenberg et al. 2001), this issue has not yet been studied thoroughly across adolescence. Thus, in the current study, we addressed this aspect, examining whether the pattern of longitudinal associations between prosocial behavior and the dimensions of empathy differed for adolescent boys and girls.

## Aims and Hypotheses

The first aim of the current study was to investigate the development of prosocial behavior between age 13 and age 18 years. Given the mixed findings of previous studies, we explored the mean level development of prosocial behavior across adolescence without making firm hypotheses. However, based on theory and results of the few previous empirical studies, we expected that gender would moderate the developmental pattern. The second aim of this study was to examine the longitudinal links between perspective taking, empathic concern, and prosocial behavior. We hypothesized that empathic concern would positively predict prosocial behavior. We also expected perspective taking to predict prosocial behavior, although this might mainly be an indirect link through empathic concern. Conversely, we hypothesized prosocial behavior to predict subsequent perspective taking and empathic concern. Finally, we explored whether the strength of the longitudinal links between perspective taking, empathic concern and prosocial behavior varied between boys and girls.

## Method

### Participants and Procedure

The present 6-wave longitudinal study used data from the ongoing RADAR (Research on Adolescent Development and Relationships) project. The “RADAR Young” cohort consisted of 497 adolescents (43% girls), who were recruited from randomly selected schools in the province of Utrecht and four cities in The Netherlands. At first measurement (in 2005), the adolescents were in their 1st year of Junior High school (*M*_age = _13.03, SD* = *0.46). Most adolescents were native Dutch (95%), lived with both parents (86%), and came from families classified as medium or high SES (89%). Most adolescents were native Dutch (95%), lived with both parents (86%), and came from families classified as medium or high SES (89%). Although the sample was drawn from the general Dutch population, due to the inclusion criteria (e.g., good understanding of written Dutch language) families in the sample differed on some characteristics from the population (Van Lier et al. 2008). That is, at the time of the data collection, 15% of the adolescents in the general population was from non-western ethnic minorities (CBS 2017a), 74% lived with both parents (Van Gaalen and Stoeldraijer 2009) and 13% of the children lived in families classified as low SES (CBS 2017b).

Annual home visits, with 1-year time intervals, were paid to the participating families, during which adolescents (and their family members) filled out a battery of questionnaires. Trained research assistants provided verbal instructions in addition to written instructions that accompanied the questionnaires. Adolescents received 20 Euros for their participation in each of the home visits. Parents were required to provide written informed consent before adolescents participated in the study. The “RADAR Young” study has been approved by the Medical Ethical Committee of Utrecht University Medical Centre (The Netherlands).

Of the original sample, 425 adolescents (86%) were still involved in the study at Wave 6, and the average participation rate over the 6 waves was 90%. Results of Little’s MCAR test revealed a normed *χ² (χ²/df)* value of 1.15 for boys and 1.11 for girls, indicating that the data were likely missing at random (Bollen 1989). Therefore, all 497 cases could be included in the analyses using a Full Information Maximum Likelihood procedure in M*plus* (Muthén and Muthén 1998–2017).

### Measures

#### Prosocial behavior

Prosocial behavior was assessed using the 11-item subscale “prosocial behavior” from the Revised Self-Report of Aggression and Social Behavior Measure (Morales and Crick 1998, 1999, reported by Linder et al. 2002). Sample items are “I’m willing to lend money to others if they really need it” and “I try to get others involved in group activities”. Adolescents rated the items on a 7-point scale, ranging from 1 (*not at all true*) to 7 (*very true*). Cronbach’s alpha reliabilities from age 13 to age 18 ranged from *α* = .90 to *α* = .93 for the total sample, from *α* = .88 to *α* = .92 for boys, and from *α* = .90 to *α* = .94 for girls. In addition to high internal consistency, previous research revealed high convergent validity for this measure (Hawk et al. 2013; Clark et al. 2015).

#### Empathy

We used two 7-item subscales of the Dutch version (Hawk et al. 2013) of the Interpersonal Reactivity Index (IRI; Davis 1983) to assess adolescents’ perspective taking (PT) and empathic concern (EC). A sample item of the PT subscale is “I try to look at everybody’s side of a disagreement before I make a decision”, and a sample item of the EC subscale is “I often have tender, concerned feelings for people less fortunate than me”. Adolescents scored the items on a 5-point scale, ranging from 0 (*doesn’t describe me at all)* to 4 (*describes me very well)*. The Dutch version of the IRI has adequate internal consistency and validity (Hawk et al. 2013). On PT, Cronbach’s alpha reliabilities from age 13 to age 18 were for the total sample, respectively, *α* = .59, *α* = .66, *α* = .77, *α* = .76, *α* = .78, and *α* = .76, for boys: *α* = .56, *α* = .56, *α* = .71, *α* = .70, *α* = .72, and *α* = .72, and for girls: *α* = .62, *α* = .71, *α* = .77, *α* = .79, *α* = .82, and *α* = .81. On EC, Cronbach’s alpha for the total sample was *α* = .62 at age 13 and ranged from *α* = .72 to *α* = .77 from age 14 to age 18. For boys, Cronbach’s alpha on EC was *α* = .58 at age 13, and ranged between *α* = .65 and *α* = .73 from age 14 to age 18. For girls, it was *α* = .60 at age 13, and ranged between, *α* = .69 and *α* = .74 from age 14 to age 18.

### Statistical Analytic Approach

First, as a preliminary step, we tested whether adolescents’ self-reports of prosocial behavior, empathic concern, and perspective taking showed longitudinal measurement invariance (Little 2013; Van de Schoot et al. 2012). For each of the three constructs, we composed a model consisting of six latent variables (one for each measurement wave) and three observed indicators for each latent variable. The indicators were constructed using the item parceling method (Little et al. 2002). Details of the parceling solution for prosocial behavior can be found in Crocetti et al. (2016) and for empathic concern and perspective taking in Hawk et al. (2013). We started with a baseline model (M1), testing configural invariance. Then, we compared the configural model with the metric model (M2) in which factor loadings are constrained to be equal across time. Since metric invariance is required for reliably examining over time associations between variables (Little 2013), we conducted this test for each of the three constructs that were included in the longitudinal path analyses (i.e., prosocial behavior, empathic concern, perspective taking). In addition, since (partial) scalar invariance is required for making meaningful mean comparisons (Byrne et al. 1989), we composed a third model for prosocial behavior in which indicator intercepts were constrained across time (M3). If full scalar invariance could not be established, we tested for partial scalar invariance (M4), constraining two out of three indicator intercepts to be equal across time (Byrne et al. 1989).

Analyses were conducted in MPlus 8 (Muthén and Muthén 1998–2017), using the Maximum Likelihood Robust estimator (MLR). We used three goodness-of-fit indices: the comparative fit index (CFI), the root mean-square error of estimation (RMSEA), and the standardized root mean-square residual (SRMR), with CFI > .90, RMSEA < .08, and SRMR < .08 indicating adequate model fit (Byrne 2012; Chen 2007; Cheung and Rensvold 2002), We examined changes in fit indices to test if the various levels of invariance could be established. ΔCFI ≥ −.01, ΔRMSEA ≥ .015, and ΔSRMR ≥ .030 (Chen 2007; Cheung and Rensvold 2002; Little 2013). If at least two out of three indices were below its threshold, invariance was assumed.

Second, to examine developmental trajectories in prosocial behavior, we used latent growth curve modelling (LGM). A model with two latent factors (i.e., intercept and linear change), a model with three latent factors (i.e., intercept, linear, and quadratic change) and a model with four latent factors (i.e., intercept, linear, quadratic, and cubic change) were compared to determine which growth curve best captured observed changes, as indicated by results of Satorra and Bentler’s (2001) scaled chi-square difference tests (S-Bχ^2^).

Third, we constructed a cross-lagged panel model to examine longitudinal bidirectional associations between perspective taking, empathic concern, and prosocial behavior. A stepwise procedure was followed to determine the best fitting, but most parsimonious, model. We started with a baseline model, which included all within time correlations between variables, and the 1-year and 2-year stability paths for all variables to establish a baseline model with acceptable fit to the data. In this baseline model, within time correlations and stabilities were constrained across time if this did not significantly worsen the model fit, indicated by results of S-Bχ^2^ tests. Subsequently, we separately added series of cross-paths, and tested whether adding these cross-paths resulted in a better model fit compared to the baseline model. The final model included stability paths, within-time correlations, and only the series of cross-paths that were found to improve the model fit. Cross-paths were constrained across time if this did not result in a significantly worse model fit. Longitudinal indirect effects were tested using the command “model indirect” in M*plus*. using maximum likelihood estimator (ML) and bootstrapping, with 95% confidence intervals based on 1000 random samples, to account for non-normality of the indirect effects (Preacher and Hayes 2008).

A multi-group approach was used in all analyses to test whether gender moderated growth and/or longitudinal links. Models in which specific parameters were constrained to be equal across the two gender groups were compared to a model in which these parameters were free to vary. The parameter was assumed to differ between boys and girls if the results of the S-Bχ^2^ test indicated the constrained model to fit significantly worse than did the model in which the parameter was free to vary across gender groups (Kline 2005).

## Results

### Preliminary Analyses

Results of longitudinal measurement invariance tests (Table 1) revealed that configural and metric invariance could be established for prosocial behavior, empathic concern, and perspective taking, as required for analyses on longitudinal links. For prosocial behavior, partial scalar invariance was established, as is required for examining mean level changes.

**Table 1:** Results of longitudinal measurement invariance tests

	Model fit						Model comparisons			
	*χ* ^2^	*df*	scaling	CFI	SRMR	RMSEA [90% CI]	Models	ΔCFI	ΔRMSEA	ΔSRMR
*Prosocial behavior*										
M1. Configural	77.678	75	1.174	.999	.027	.008 [.000, .027]				
M2. Metric	90.558	85	1.202	.999	.036	.011 [.000, .028]	M2-M1	.000	.003	.009
M3. Scalar	164.540	100	1.191	.985	.061	.036 [.026, .046]	M3-M2	−.014	.025	.025
M4. Partial Scalar	133.628	95	1.193	.991	.054	.029 [.016, .039]	M4-M2	−.008	.018	.018
*Empathic concern*										
M1. Configural	89.853	75	1.134	.995	.025	.020 [.000, .034]				
M2. Metric	99.553	85	1.135	.995	.032	.019 [.000, .032]	M2-M1	.000	−.001	.007
*Perspective taking*										
M1. Configural	103.319	75	1.135	.991	.052	.028 [.012, .040]				
M2. Metric	121.590	85	1.138	.989	.060	.029 [.016, .041]	M2-M1	−.002	.001	.008

### Development of Prosocial Behavior

**Fig. 1:**
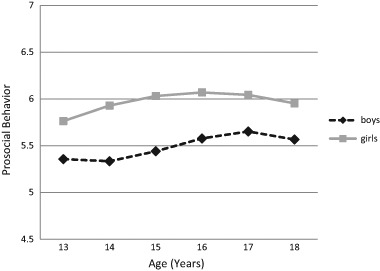
Estimated means of prosocial behavior for boys (dashed black line) and for girls (solid grey line) from age 13 to age 18

Table 2 shows the means and standard deviations for the study variables for boys and girls. To examine mean level changes in prosocial behavior, we conducted LGMs. Adding a quadratic growth term to the model improved the model fit, ΔS-Bχ^2^ (8) = 16.94, *p < *.05. Comparing quadratic and cubic models revealed the cubic model to capture growth in prosocial behavior significantly better than the quadratic model, ΔS-Bχ^2^ (2) = 12.62, *p < *.01. Multiple group analyses revealed significant gender differences in initial levels, ΔS-Bχ^2^ (1) = 14.51, *p < *.001, with boys (intercept = 5.36, *p* < .001) showing lower levels of prosocial behavior than did girls (intercept = 5.76, *p* < .001). Moreover, gender also moderated the developmental pattern, ΔS-Bχ^2^ (3) = 10.56, *p < *.05 (see Fig. 1). Since the cubic growth term appeared significant for boys (*p < *.01) but far from significant for girls (*p = *.83), the cubic growth term was included for boys only. The final model, in which all growth parameters were free to vary between boys and girls, showed good fit to the data, S-Bχ^2^ (22, *N = *497) = 26.59, *p = *.27, CFI = .98, RMSEA = .03. For boys, stable levels between age 13 and 14 were followed by an increase until age 17, and a slight decrease thereafter (linear slope = −.12, *p = *.15, quadratic slope = .11, *p < *.01, cubic slope = −.02, *p < *.01). For girls, prosocial behavior increased until age 16 with a slight decrease thereafter (linear slope = .20, *p < *.001, quadratic slope = −.03, *p < *.001).

**Table 2:** Means and standard deviations of boys’ and girls’ prosocial behavior, empathic concern, and perspective taking

		Age 13		Age 14		Age 15		Age 16		Age 17		Age 18	
		*M*	*SD*	*M*	*SD*	*M*	*SD*	*M*	*SD*	*M*	*SD*	*M*	*SD*
*Prosocial behavior*													
Total		5.53	0.93	5.60	1.02	5.71	0.89	5.75	0.88	5.86	0.81	5.77	0.90
Boys		5.36	0.90	5.34	0.98	5.48	0.90	5.52	0.90	5.70	0.72	5.64	0.87
Girls		5.76	0.92	5.96	0.96	6.02	0.79	6.07	0.75	6.05	0.87	5.93	0.92
*Empathic concern*													
Total		2.46	0.55	2.46	0.62	2.44	0.65	2.39	0.66	2.45	0.58	2.47	0.60
Boys		2.32	0.54	2.25	0.57	2.18	0.57	2.16	0.63	2.26	0.54	2.27	0.56
Girls		2.65	0.51	2.74	0.57	2.77	0.59	2.69	0.57	2.70	0.54	2.74	0.55
*Perspective taking*													
Total		2.05	0.53	2.11	0.58	2.11	0.63	2.18	0.62	2.22	0.63	2.29	0.60
Boys		2.01	0.51	2.00	0.51	1.94	0.58	2.03	0.60	2.11	0.58	2.18	0.57
Girls		2.10	0.54	2.27	0.63	2.33	0.63	2.37	0.60	2.37	0.65	2.44	0.62

### Longitudinal Links between Perspective Taking, Empathic Concern, and Prosocial Behavior

The baseline model, including 1- and 2-year stabilities of perspective taking, empathic concern, and prosocial behavior, and including the within-time correlations between these variables, showed acceptable model fit, S-Bχ^2^ (275, *N = *497) = 442.76, *p < *.001, CFI = .93, RMSEA = .05. Regarding the prediction of prosocial behavior, adding the cross-paths from empathic concern to prosocial behavior significantly improved model fit, ΔS-Bχ^2^ (5) = 23.64, *p < *.001, whereas adding cross-paths from perspective taking to prosocial behavior did not significantly improve model fit (*p = *.10). Regarding the reversed cross-paths, adding the cross-paths from prosocial behavior to empathic concern significantly improved model fit, ΔS-Bχ^2^ (5) = 13.74, *p < *.05, but adding the cross-paths from prosocial behavior to perspective taking did not (*p = *.23). In addition, adding the cross-paths from perspective taking to empathic concern improved the model fit, ΔS-Bχ^2^ (5) = 25.96, *p < *.001, as did the reversed cross-paths, from empathic concern to perspective taking, ΔS-Bχ^2^ (5) = 25.17, *p < *.001.^1^ All cross-paths could be constrained across time without worsening the model fit. Regarding gender differences, only for the cross-paths from prosocial behavior to empathic concern, the model fit improved significantly if the parameters were allowed to vary between boys and girls, ΔS-Bχ^2^ (1) = 7.63, *p < *.01.^2^ The final model, including 1- and 2-year stability paths, within time correlations (see Table 3), cross-paths from empathic concern to prosocial behavior and vice versa, and cross-paths from perspective taking to empathic concern and vice versa, showed good fit to the data, S-Bχ^2^ (270, *N = *497) = 353.514, *p < *.001, CFI = .97, RMSEA = .04. The percentage explained variance in prosocial behavior ranged for boys between 8.9–27.9%, and for girls between 12.0–27.5%, in empathic concern for boys between 18.6–44.4%, and for girls between 22.0–48.8%, and in perspective taking it ranged for boys between 21.7–45.7%, and for girls between 16.1–44.5%.

**Fig. 2:**
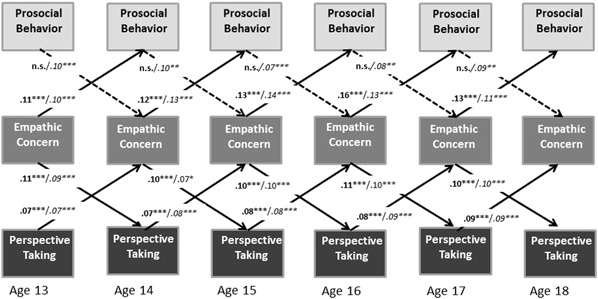
Standardized estimates of cross-lagged paths for boys (printed bold) and for girls (printed italic). Black arrows represent paths that are significant for both boys and girls, dashed arrows represent paths that are significant for girls only. Although not displayed, this model includes within-time correlations and 1- and 2-year stability paths. **p < .01; ***p < .001

Figure 2 depicts the cross-lagged results from the final model, showing that higher empathic concern significantly predicted higher prosocial behavior for boys and girls at all ages. Conversely, higher prosocial behavior significantly predicted higher empathic concern at all ages, but only for girls. For boys, the paths from prosocial behavior to empathic concern were not significant. Further, for both boys and girls, higher empathic concern significantly predicted higher perspective taking, and higher perspective taking significantly predicted higher empathic concern, at all ages. Although there were no direct cross-lagged effects between perspective taking and prosocial behavior, higher perspective taking did predict higher prosocial behavior indirectly via empathic concern for boys and girls (*b = *.015, 95% CI = .009, .024). Further, within-time correlations indicated that higher empathic concern was related to higher prosocial behavior at all ages for boys and girls, and higher perspective taking was related to higher prosocial behavior at all ages, except Age 16 for boys and girls.

## Discussion

The tendency to engage in prosocial behavior is thought to be subject to change during adolescence. Yet, evidence from previous research regarding the direction of this change is inconsistent: increases (e.g., Eisenberg et al. 2005), as well as, decreases (e.g., Carlo et al. 2007) and stability (e.g., Nantel‐Vivier et al. 2009) have been reported. These contrasting results suggest that the development of prosocial behavior may follow a complex developmental pattern during adolescence, which could not be captured in the relatively short age span of adolescent years that mainly has been used in previous studies (e.g., Caprara et al. 2015; Nantel-Vivier). Therefore, the first aim of the current six-wave longitudinal study was to investigate mean-level development in prosocial behavior between ages 13 and 18 years, and to take potentially complex and gender-specific growth patterns into account. The second aim of this study was to investigate the longitudinal links of both empathic concern and perspective taking with prosocial behavior across adolescence. Both are generally deemed important predictors of prosocial behavior (Batson 1991; Hoffman 2000), but studies that have investigated the links of both constructs with prosocial behavior are sparse. Moreover, since most of the research on this topic has been cross-sectional, the potentially reciprocal nature of the associations hasn’t been taken into account yet. Therefore, we investigated the bidirectional links of empathic concern and perspective taking with prosocial behavior across adolescence.

Regarding the first aim of this study, we indeed found increases in prosocial behavior between early and mid-adolescence, which is in line with the notion that physical, cognitive, and relational advances foster adolescents’ other-oriented behavior. This result is consistent with previous findings of increasing prosocial behavior between age 13 and 16 years (Carlo et al. 2015) and between age 15 and 17 years (Eisenberg et al. 2005). Our result is distinct, however, from the previously reported decrease in prosocial behavior in mid-adolescence (Carlo et al. 2007). These mixed findings may be due to differences in the sample characteristics. For example, in contrast to the present sample of Dutch adolescents from primarily urban areas and from families of medium to high level SES, the Carlo et al. (2007) sample consisted of adolescents from primarily rural communities and from families of relatively low SES in the United States. Prosocial behavior in rural areas may be relatively low as a result of depleted social capital and community resources (Carlo et al. 2007) and youth from low SES families may have limited opportunities to develop their tendency to show prosocial behavior, because they are not often involved in structured and adult-supervised activities (Hart and Atkins 2002). Given previous evidence of significant links between SES and prosocial behavior (Eisenberg et al. 2006), these findings underline the need for future research to examine in more diverse samples whether age trends in prosocial behavior differ between adolescents from high and low SES families, and from urban and rural communities. If differences in the age-related trends between studies are partly due to SES differences, then the findings suggest efforts to enrich low-SES communities with activities and resources that provide prosocial behavior opportunities.

Of additional interest is the finding that the developmental trend in prosocial behavior was gender-specific. Consistent with previous research, boys reported lower levels of prosocial behavior than girls (e.g., Carlo et al. 2015; Crocetti et al. 2016; Eisenberg et al. 2005), but whereas girls’ prosocial behavior increased between age 13 and age 16 years, boys’ prosocial behavior increased between age 14 and age 17 years. This finding may reflect gender differences in cerebral cortical development. During early adolescence, girls undergo a faster acceleration in cerebral cortical development than boys (Andrich and Styles 1994; Colom and Lynn 2004), and therefore girls are generally about 2 years ahead of boys in intellectual and social-cognitive functioning until mid-adolescence (Silberman and Snarey 1993; Porteous 1985). Furthermore, as a result of the different timing of the increase for boys and girls, the gender difference in mean levels of prosocial behavior was largest in mid-adolescence. This result is in line with gender role intensification theory, which suggests that the adherence to gender role expectations is particularly strong during mid-adolescence (Alfieri et al. 1996; Fabes et al. 1999; Hill and Lynch 1983), and is also consistent with the previous finding of increasing gender differences in levels of empathic concern and perspective taking between early- and mid-adolescence (Van der Graaff et al. 2014). However, prosocial behavior showed a decrease between age 16 and 18 years for girls, and between age 17 and 18 years for boys (Carlo et al. 2007; Luengo Kanacri et al. 2013). An explanation for this finding may be that Dutch adolescents usually finish high school, start a study either at a university or a vocational school, and/or have their first paid job between age 16 and 18 years. Such changes in adolescents’ lives and roles may lead them to focus (temporarily) on their own rather than others’ needs.

Interestingly, across adolescence, mean-level changes in prosocial behavior showed a quadratic pattern for girls, and a cubic pattern for boys. This finding is in accordance with previous studies that demonstrated complex patterns of age-related changes during adolescence as well (e.g., Carlo et al. 2007; Eisenberg et al. 2005). Moreover, the fact that growth in prosocial behavior appears to be gender-specific and non-linear may also explain the inconsistencies between previous studies that investigated prosocial behavior during different stages of adolescence. For instance, the findings of stable levels between age 12 and 14 years (Nantel‐Vivier et al. 2009), increasing levels between age 13 and 16 years (Carlo et al. 2015), and non-linear increases between age 12 and 14 years (Caprara et al. 2015) all fit within the findings of the current study showing that levels of prosocial behavior are stable for boys between age 13 and 14 years, increase for both boys and girls until age 16, and slightly decrease thereafter.

**Table 3:** Standardized 1-year and 2-year stabilities, and standardized within-time correlations between prosocial behavior (PB), empathic concern (EC), and perspective taking (PT) for boys (below diagonal) and for girls (above diagonal)

	1.	2.	3.	4.	5.	6.	7.	8.	9.	10.	11.	12.	13.	14.	15.	16.	17.	18.
1. PB age 13	–	.32***	.17**				.40***						.27***					
2. PB age 14	.31***	–	.18**	.17**				.22***						.10*				
3. PB age 15	.14**	.15**	–	.29***	.16**				.28***						.24***			
4. PB age 16		.14*	.29***	–	.17**	.12**				.20***						.10		
5. PB age 17			.23***	.25***	–	.43***					.21***						.18***	
6. PB age 18				.15*	.39***	–						.15**						.16***
7. EC age 13	.42***						–	.38***	.20***				.50***					
8. EC age 14		.23***					.39***	–	.42***	.21***				.35***				
9. EC age 15			.23***				.20***	.42***	–	.39***	.23***				.50***			
10. EC age 16				.18**				.21***	.41***	–	.46***	.24***				.39***		
11. EC age 17					.27***				.23***	.46***	–	.42***					.34***	
12. EC age 18						.18**				.25***	.43***	–						.27***
13. PT age 13	.30***						.48***						–	.35***	.21***			
14. PT age 14		.13*						.40***					.40***	–	.40***	.25***		
15. PT age 15			.22***						.47***				.22***	.36***	–	.40***	.25***	
16. PT age 16				.09						.39***				.22***	.40***	–	.40***	.26***
17. PT age 17					.26***						.35***				.25***	.41***	–	.42***
18. PT age 18						.20***						.29***				.26***	.42***	–

*p < .05; **p < .01; ***p < .001

Regarding the second aim of this study, consistent with posited models of prosocial development (Batson 1991; Eisenberg and Miller 1987; Hoffman 2000), we found that empathic concern was longitudinally related to subsequent prosocial behavior for both boys and girls. Moreover, perspective taking was indirectly related to later prosocial behavior, via its effect on empathic concern. These findings are in accord with scholars’ assertions regarding the central role of empathic concern in predicting prosocial behavior (Batson et al. 1989; Eisenberg et al. 2001). Indeed, perspective taking did not predict prosocial behavior directly, which affirms the importance of empathic concern rather than perspective taking as a relatively stronger predictor of such actions and is consistent with prior research on the mixed relations between perspective taking and prosocial behavior (see Carlo et al. 2010a, for a meta-analytic review). On the other hand, the present findings showed that perspective taking still plays an indirect role, by its longitudinal association with empathic concern, which in turn was related to subsequent prosocial behavior. Indeed, there were bidirectional relations between perspective taking and empathic concern across all ages and for both genders. This result is conflicting however, with the finding of a previous study that showed perspective taking not to predict later empathic concern (Van Lissa et al. 2014). This may be explained by the use of latent constructs in that study, resulting in high rank-order stability of empathic concern. The findings of the current study showing consistent bidirectional relations between perspective taking and empathic concern and indirect effects of perspective taking on later prosocial behaviour are consistent with moral development theories (Eisenberg et al. 2006; Hoffman 2000) that highlight the interplay of perspective taking and empathic concern in the prediction of prosocial behavior,

Because no prior research examined the bidirectional relations among prosocial behavior, perspective taking, and empathic concern, these relations are of particular interest. The present study reveals limited evidence for prosocial behavior as a predictor of empathic concern; prosocial behavior was associated with later empathic concern, but only for girls. In contrast, earlier prosocial behavior was not related with subsequent perspective taking. The former set of findings suggests that adolescence may be a particularly sensitive period for the development of prosocial traits for girls relative to boys perhaps as a result of social feedback on overt expressions of empathic concern. Gender differences in empathic concern are relatively consistent with gender stereotypes regarding the expression of such emotions in girls (Brody 1999; Hoffman 1977). A previous study demonstrated increasing gender differences in empathic concern, favouring girls, between early and mid-adolescence (Van der Graaff et al. 2014). The present findings support the notion that the interplay between empathic concern and prosocial behavior may have a stronger reinforcing quality for girls relative to boys. This latter explanation is in accord with scholars who note that early to middle adolescence is an age period of gender intensification, whereby boys and girls are subjected to strong pressures to conform to gender-type behaviors (Fabes et al. 1999). Therefore, the gender-related findings may result from stronger gender-consistent stereotyped notions of prosocial behaviors as feminine-acceptable actions (Carlo et al. 2012; Eagly and Crowley 1986).

Despite the relatively large sample and the cross-lagged design, there were some study limitations. First, the measures of prosocial behavior were adolescents’ self-reports, which raises concerns regarding shared method variance and self-presentational demands. Future research using multiple methods (e.g., observational) and/or multiple reporter (e.g., peer ratings) measures is desirable to reduce such concerns. And second, the sample is relatively homogenous and the findings may not generalize to broader or more diverse (e.g., across SES, ethnicities) populations of adolescents. Future studies that include larger and more representative youth samples might better address possible moderating effects of other demographic variables (e.g., SES, ethnicity). Nonetheless, the present findings significantly extend our understanding of age-related changes in prosocial behavior and in the links among sociocognitive and socioemotive traits, and prosocial behavior across adolescence.

## Conclusion

The present study yields evidence suggesting that prosocial behavior increases until mid-adolescence, and slightly decreases thereafter. Moreover, our results underscore that the development of prosocial behaviour during adolescence is gender-specific: growth in prosocial behavior starts earlier for girls than for boys, and, in accordance with gender role intensification theory, gender differences increase between early and mid-adolescence (Hill and Lynch 1983). The complex and gender-specific growth patterns as found in this comprehensive study may explain the inconsistencies between previous studies that investigated shorter age spans during adolescence and/or did not take gender differences in developmental patterns into account. Our finding that prosocial behavior increases during mid-adolescence, is in line with the notion that adolescents’ physical maturity, increasing autonomy and cognitive advances (which come earlier for girls than for boys) facilitate the tendency to engage in prosocial behavior (Carlo et al. 2012; Fabes et al. 1999). The slight decreases in later adolescence may result from changes in adolescents’ roles and lives as they move away from their familiar surroundings and start attending college or having their first job. The current study also demonstrated reciprocal relations between empathic concern and prosocial behavior (especially for girls), and mediating effects of empathic concern in the relations between perspective taking and prosocial behavior. Moreover, there were no direct over time effects of perspective taking on prosocial behavior. These findings suggest that moral emotions may be relatively more intractably tied to prosocial behavior than moral cognitions; though moral cognitions can still play an important role in fostering moral emotions and specific forms of prosocial behavior (e.g., those that require social understanding, cost-benefit analyses, or reasoning). Future research that examines the age- and gender-related correlates of adolescents’ prosocial behavior and the conditions under which moral cognitions and moral emotions predict such actions is needed. The current results suggest that prevention and intervention strategies should focus on promoting empathic concern among adolescents to facilitate growth in prosocial behavior. In addition, promoting adolescents’ perspective taking may be beneficial as well, as it facilitates empathic concern, which in turn stimulates helping behavior. Finally, given the pattern of gender-related findings, such efforts may need to be modified for boys and girls to enhance their effectiveness.

## Footnotes

1 Including all series of crosspaths in the model, instead of using a model building approach, revealed the same results, with significant crosspaths between empathic concern and prosocial behavior, significant crosspaths between empathic concern and perspective taking, but insignificant crosspaths between perspective taking and prosocial behavior.

2 We arrived at the same model (including all crosspaths except those between perspective taking and prosocial behavior) if estimates were allowed to vary between boys and girls when testing if adding a series of crosspaths to the baseline model improved model fit.
